# Phytochemistry and Anticancer Effects of Mangrove (*Rhizophora mucronata* Lam.) Leaves and Stems Extract against Different Cancer Cell Lines

**DOI:** 10.3390/ph16010004

**Published:** 2022-12-20

**Authors:** Ahmed M. M. Youssef, Doaa A. M. Maaty, Yousef M. Al-Saraireh

**Affiliations:** 1 Department of Pharmacology, Faculty of Pharmacy, Mutah University, P.O. Box 7, Al-Karak 61710, Jordan; 2 Department of Botany and Microbiology, Faculty of Science, Al-Azhar University, Girls Branch, Cairo 11754, Egypt; 3 Department of Pharmacology, Faculty of Medicine, Mutah University, P.O. Box 7, Al-Karak 61710, Jordan

**Keywords:** alkaloids, cancers, cytotoxicity, flavonoids, phytochemistry, *Rhizophora mucronata*, steroids

## Abstract

The biologically active components of the methanol extracts of *R. mucronata* were identified using GC/MS. The anticancer effects of each methanol extract from the leaves and stem were evaluated against cancer and non-cancer cell lines. The MTT assay was used in order to evaluate cell viability, and the IC_50_ and the selectivity indices were calculated in relation to a positive control (doxorubicin). The results showed that 11 and 8 different chemical compounds were found in the methanol extracts from the leaves and stems of *R. mucronata*, respectively. The active constituents of *R. mucronata* leaves and stems had anticancer effects against colon cancer (CaCo-2), with IC_50_ levels of 127 ± 4 μg/mL and 107 ± 6 μg/mL, respectively, and on breast cancer (MCF-7), with IC_50_ levels of 158 ± 10 μg/mL and 138 ± 4 μg/mL, respectively. These were both greater than their effects on prostate cancer (PC-3), for which they showed IC_50_ levels of 480 ± 14 μg/mL and 294 ± 3 μg/mL, respectively. However, the anticancer effect of the stems on lung cancer (A549) (IC_50_ = 155 ± 10 μg/mL) was greater than that of the leaves (IC_50_ = 376 ± 9 μg/mL) in comparison with doxorubicin. Neither the stems nor the leaves of *R. mucronata* showed any cytotoxicity against normal cells (WI-38), with the IC_50_ being 932 ± 30 μg/mL for the leaves and 629 ± 3 μg/mL for the stems.

## 1. Introduction

Chemotherapy remains the main treatment approach for cancer patients, particularly for those in the late stages of disease. Nevertheless, the development of drug resistance and severe adverse effects constrain the use of chemotherapy for cancer therapy [1]. The emergence of these chemotherapy problems is mainly due to drug inactivation and metabolic biotransformation by several enzymes, such as cytochromes P450 [2,3]. Identifying the molecular mechanisms that drive these problems remains a significant field of research that can help to identify novel pharmacological drug targets and to discover new drug leads, particularly from natural products, in order to improve patients’ therapeutic outcomes [4,5,6,7]. Recent scientific and technological developments have opened up new opportunities to better address the potential of natural-product-based drug discovery for cancer treatment. Nowadays, there is increasing interest in natural products as pharmacological drug leads, particularly to tackle cancer resistance [4,8,9,10]. Therefore, joint efforts must be made in order to identify novel natural-product-based cancer therapies.

*Rhizophora* L. (Linnaeus, Carl) is the only genus of the Rhizophoraceae family in Egypt. It is represented by only one species: *Rhizophora mucronata* Lam. (Lamarck, De) [11]. Along with Avicennia marina, it forms a certain type of vegetation in tidal saline wetlands and grows in scattered patches along the southern extension of the Red Sea coast of Egypt [11]. The traditional use of *R. mucronata* has recently been reviewed by Patra and Thatoi [12]. The most common traditional uses of *R. mucronate* are as an antipyretic and in the treatment of elephantiasis, hematoma, hepatitis, and ulcers [13,14]. It also possesses antiviral [15,16], antibacterial [17], antioxidant [18], and anti-inflammatory properties [19]. Additionally, the fruit flour of ripe *R. mucronata* may be used for diabetic patients [20], and its leaves may be used as an antidiarrheal medication [21]. *R. mucronata* contains several biologically active compounds, such as phenolics, alkaloids, steroids, saponins, flavonoids, tannins, triterpene, and diterpenoids [22]. *R. mucronata* has been studied in vitro and has proven to be able to inhibit the growth of cancer cells, including cervical [23], breast [24], and colon cancer cells [25]. The objective of this work is to identify and evaluate the possible anticancer activities of the biological substances present in the methanol extracts obtained from the leaves and stems of *R. mucronata.*

## 2. Results

### 2.1. Phytochemistry

**Figure 1:**
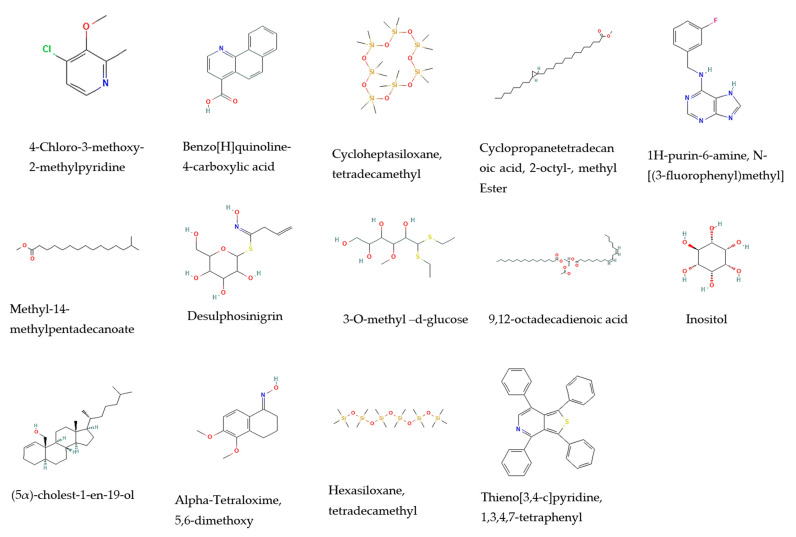
The chemical structures of the chemical compounds identified from methanol extracts from the leaves and stems of *Rhizophora mucronata* via GC/MS.

**Figure 2:**
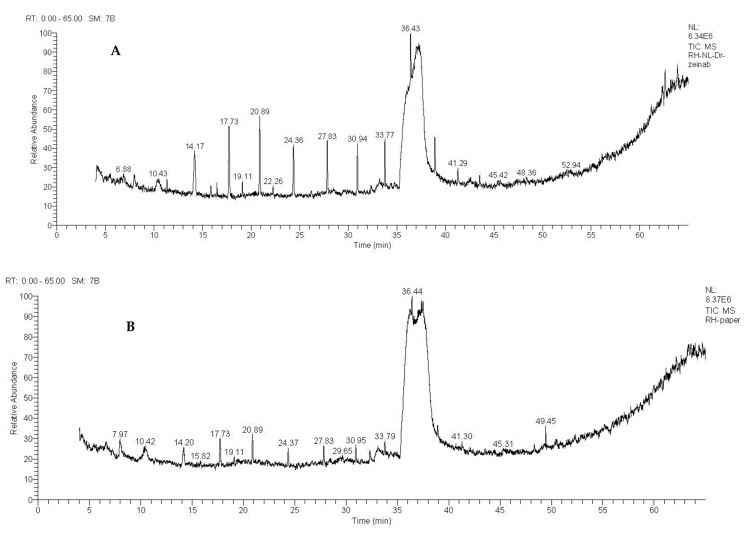
Gas chromatography/mass spectrometry spectra of *Rhizophora mucronate*. (**A**) Stem extract. (**B**) Leaf extract.

The gas chromatography/mass spectrometry analysis of the methanolic extracts from the leaves and stems of *R. mucronata* were found to contain different bioactive compounds (Table 1, Figure 1 and Figure 2). The major components of the methanol extracts from both the leaves and stems were inositol (relative abundance (RA) = 33.67% and 27.59%, respectively) and 3-O-methyl-d-glucose (RA = 6.45% and 2.63%, respectively). The spectrum showed three *m*/*z* peaks in the methanol extracts from the leaves and stems, including a quinoline alkaloid derivative, benzo[H]quinoline-4-carboxylic acid (RA = 0.67% and 0.9%, respectively); a heterocyclic aromatic compound, 1H-purin-6-amine, N- [(3-fluorophenyl)methyl] (RA = 6.10% and 7.53%, respectively); and a glucosinolate, desulphosinigrin (RA = 3.97% and 1.4%, respectively). Alkaloid, 4-Chloro-3-methoxy-2-methylpyridine (RA = 1.94%); a fatty acid derivative, cyclopropane tetradecanoic acid, 2-octyl-, methyl ester (RA = 0.61%); methyl 14 methylpentadecanoate (RA = 4.34%); a doubly unsaturated fatty acid, 9,12-octadecadienoic acid (RA = 1.14%); a one cholestane steroid, (5α)-cholest-1-en-19-ol (RA = 2.30%); and an organo-silicone compound, tetradecamethyl-cycloheptasiloxane (RA = 2.37%) were identified in the leaves. However, the constituents found in the stems were α-tetraloxime, 5,6-dimethoxy (RA = 1.14%); a hexasiloxane, tetradecamethyl (RA = 3.82%); and a pyridine alkaloid derivative, thieno[3,4-c]pyridine, 1,3,4,7-tetraphenyl (RA = 0.95%).

**Table 1:** Active principal identification of extracts from the leaves and stems of *Rhizophora mucronata* by GC/MS.

No.					Leaves		Stems		Biological Activity	References
	Compounds	MW	M.F.	Category	Rt	RA%	Rt	RA%		
1	4-Chloro-3-methoxy-2-methylpyridine	157	C7H8ClNO	Alkaloid	7.98	1.94			No data available	
2	Benzo[H]quinoline-4-carboxylic acid	223	C14H9NO2	Quinoline alkaloid	10.42	0.67	10.42	0.9	Anticancer	[26]
3	Cycloheptasiloxane, tetradecamethyl	518	C14H42O7Si7	Organo-silicone compound	14.19	2.37			Anticancer and antimicrobial	[27,28]
4	Cyclopropanetetradecanoic acid, 2-octyl-, methyl Ester	394	C26H50O2	Fatty acid	19.11	0.61			Antimicrobial	[29,30]
5	1H-purin-6-amine, N- [(3-fluorophenyl)methyl]	243	C12H10FN5	Fluorinated aromatic compound	20.89	6.10	27.8	7.53	Antioxidant	[31]
6	Methyl-14-methylpentadecanoate	270	C17H34O2	Fatty acid	32.31	4.34			Antimicrobial	[29,30]
7	Desulphosinigrin	279	C10H17NO6S	Glucosinolate	33.10	3.97	33.2	1.49	Anticancer and Antimicrobial	[32,33,34]
8	3-O-methyl-d-glucose	194	C7H14O6	D-aldohexose	35.40	6.45	35.79	2.63	Anti-inflammatory and antioxidant	[35]
9	9,12-octadecadienoic acid	280	C18H32O2	Fatty acid	33.78	1.14			Anticancer and antibacterial	[36,37]
10	Inositol	180	C6H12O6	A cyclic carbohydrate	35.99	33.67	37.36	27.59	Anticancer	[38]
11	(5α)-cholest-1-en-19-ol	386	C27H46O	Cholestane steroids	49.45	2.30			No data available	
12	Alpha-Tetraloxime, 5,6-dimethoxy	221	C12H15NO3	Aromatic-organic compound			22.26	1.14	No data available	
13	Hexasiloxane, tetradecamethyl	458	C14H42O5Si6	Linear siloxanes			24.36	3.82	Antimicrobial	[39]
14	Thieno[3,4-c]pyridine, 1,3,4,7-tetraphenyl	439	C31H21NS	Pyridine alkaloid			45.42	0.95	Anticancer	[40]

Rt: retention time; RA: relative abundance.

### 2.2. Cytotoxicity

**Figure 3:**
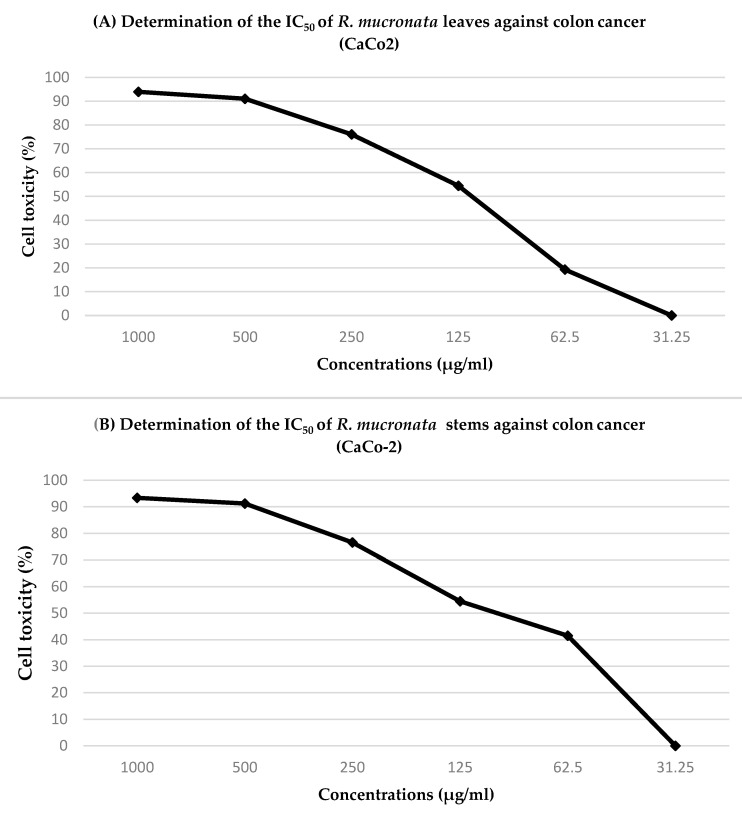
Determination of the half-maximal inhibitory concentration (IC_50_) of (**A**) *R. mucronata* leaf extract and (**B**) *R. mucronata* stem extract against colon cancer (CaCo-2).

The concentrations of the leaf and stem extracts were plotted on the *X*-axis in order to determine the half-maximal inhibitory concentration. The percentage of cytotoxicity is expressed as (cell viability % − 100) on the *Y*-axis (IC_50_). For instance, the IC_50_ of the leaf extract against colon cancer (CaCo-2) was 127 ± 4 µg/mL, whereas the IC_50_ of the stem extract against colon cancer was 107 ± 6 µg/mL (Figure 3).

Using the MTT test, the anticancer properties of *Rhizophora mucronata* leaves and stems were studied with respect to the studied cancer and non-cancer cell lines (Table 2). The Dunnett’s test was used to compare all the IC_50_ values of the leaf and stem extracts to the IC_50_ values of doxorubicin, a positive control. Based on the NCI criteria, the methanol extracts from the leaves and stems exhibited various antitumor effects on the studied cancer cell lines, as shown in Table 2. The antitumor effects of *R. mucronata* leaves and stems on prostate cancer (PC-3), with IC_50_ values of 480 ± 14 μg/mL and 294 ± 3 μg/mL, respectively, were weak compared to doxorubicin. However, the anticancer effects of the leaves and stems on colon cancer (CaCo-2), with IC_50_ values of 107 ± 6 μg/mL and 127 ± 4 μg/mL, respectively, and breast cancer, with IC_50_ values of 158 ± 10 μg/mL and 138 ± 3 μg/mL, respectively, were moderate in compared to the positive control. In contrast, the anticancer effect of the stems (IC_50_ = 155 ± 10 μg/mL) on lung cancer (A549) was higher than that of the leaves (IC_50_ = 376 ± 9 μg/mL). Importantly, the extracts from the leaves and stems did not exhibit any cytotoxicity against non-cancer cells (WI-38), with IC_50_ values of 932 ± 30 μg/mL and 629 ± 3 μg/mL, respectively, relative to the positive control. The methanol extract from *R. mucronata* stems produced selective cytotoxicity in the CaCo-2, MCF-7, and A549 cell lines. However, no cytotoxic selectivity was shown for the leaf extract in the PC-3 or A549 cell lines (values < three) (Table 3).

**Figure 4:**
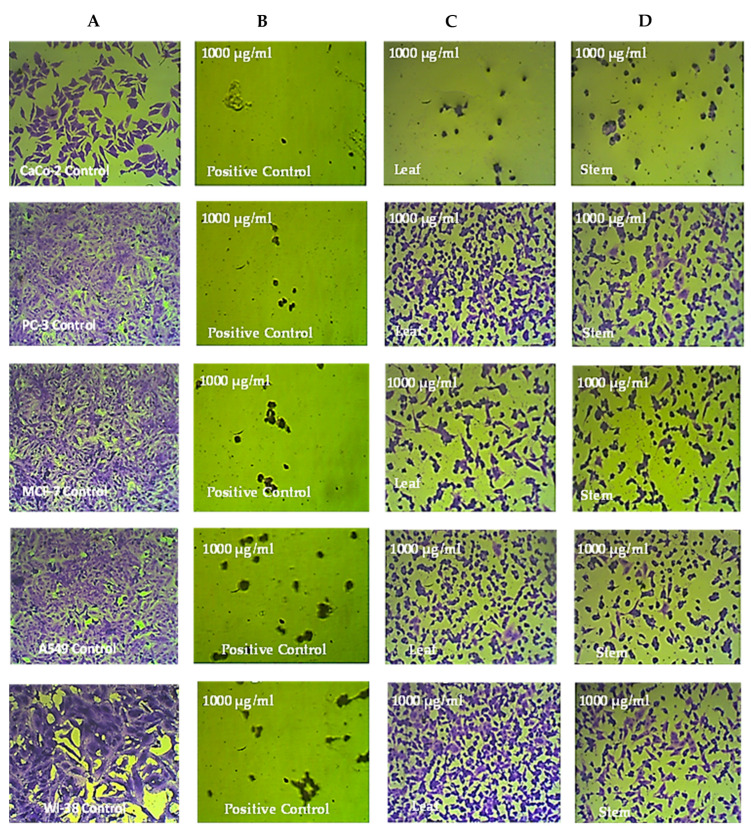
The anticancer effects of *R. micronata* stem and leaf extracts in methanol on cancer cell lines. (**A**) Complete monolayer sheets are seen in all cancer cell lines that have not been treated. (**B**) Doxorubicin treatment results in rounded and shrunken cells in all cancer cell lines. (**C**) Tiny, shrunken, and rounded cells are visible in CaCo-2, MCF-7, and A549 cell lines, as well as in PC-3 and WI-38 cell lines. (**D**) The methanol extract of *R. micronata* stem used to treat cancer cell lines revealed significantly rounded and shrunken cells in colon cancer (CaCo-2), breast cancer (MCF-7), and lung cancer (A549), as well as smaller, rounded, and shrunken cells in PC-3 and WI-38.

**Figure 5:**
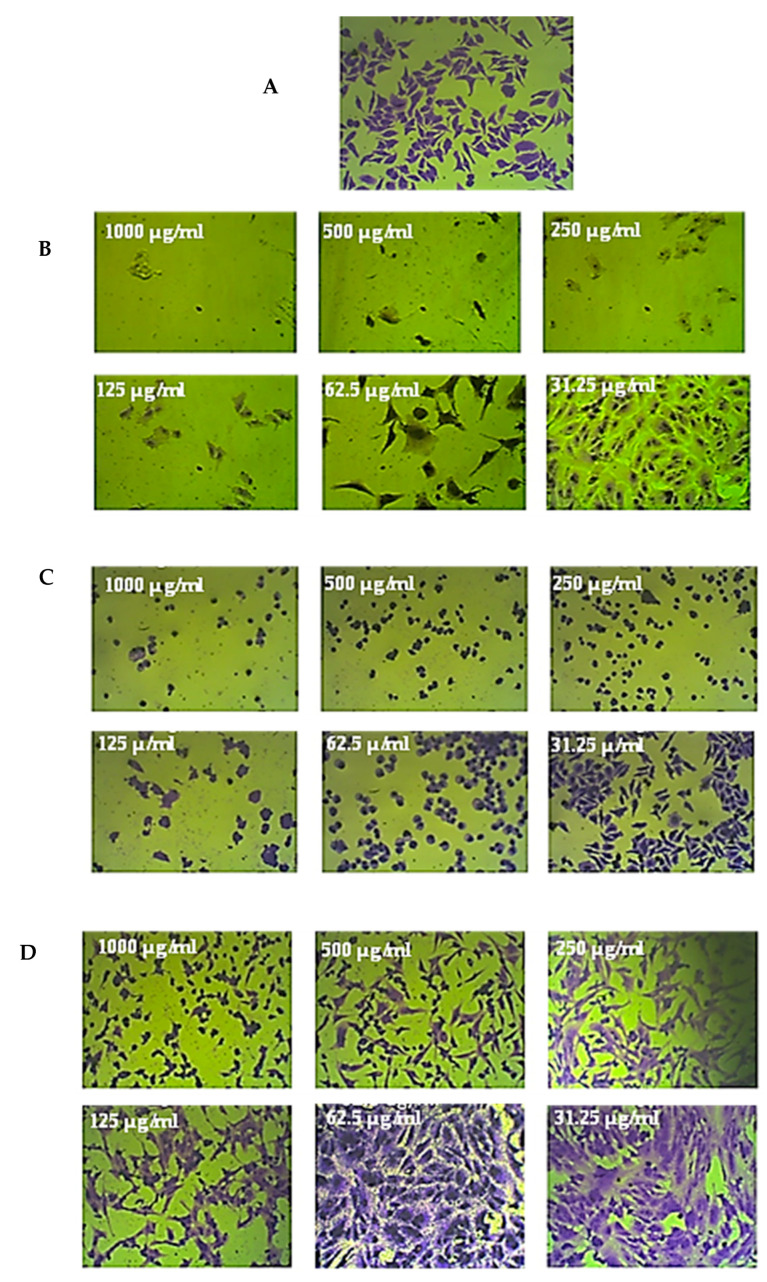
An example of the moderate anticancer effects of the stem extract of *R. micronata* against colon cancer cell lines (CaCo-2). (**A**) Complete monolayer sheets of colon cancer cell lines (CaCo-2) that have not been treated. (**B**) The effect of doxorubicin treatment at different concentrations. (**C**) The effect of the stem extract of *R. micronata* against CaCo-2 cell lines at different concentrations. (**D**) The effect of the stem extract of *R. micronata* against normal human fetal lung fibroblasts (WI-38) at different concentrations.

**Figure 6:**
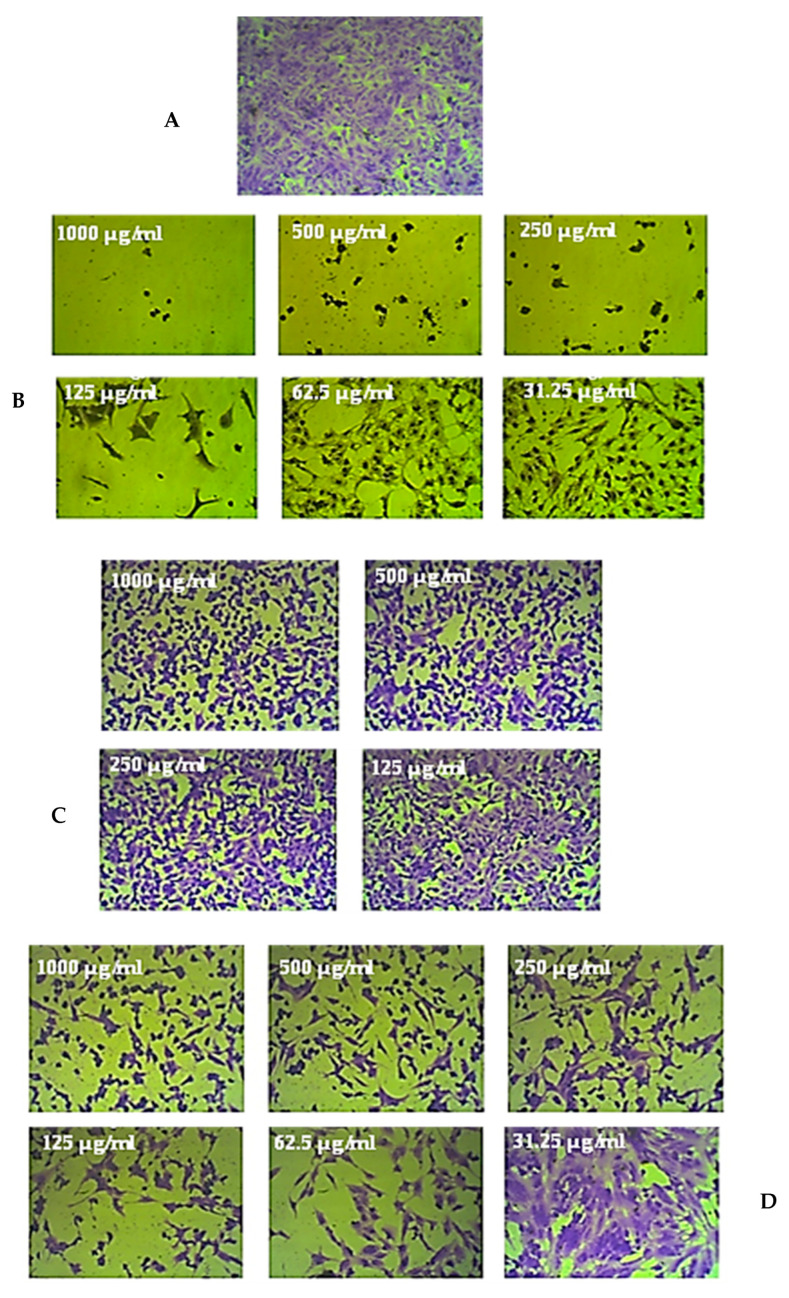
An example of the weak anticancer effects of the leaf extract of *R. micronata* against prostate cancer cell lines (PC-3). (**A**) Complete monolayer sheets of prostate cancer cell lines (PC-3) that have not been treated. (**B**) The effect of doxorubicin treatment at different concentrations. (**C**) The effect of the leaf extract of *R. micronata* against PC-3 cell lines at different concentrations. (**D**) The effect of the leaf extract of *R. micronata* against normal human fetal lung fibroblasts (WI-38) at different concentrations.

The various cell lines that were treated for 72 h with 1000 µg/mL of the leaf and stem extracts of *R. mucronata* were examined under a microscope. Compared to the untreated control cells, CaCo-2 cell lines exhibited significant shrinkage after treatment with the methanol extract from the leaves and stems of *R. mucronata*. These cells also became rounded and disconnected. However, the prostate cancer, breast cancer, lung cancer, and normal cell lines exhibited only small alterations in their morphology compared to the control cells, as shown in Figure 4, Figure 5 and Figure 6.

## 3. Materials and Methods

### 3.1. Active Constituent Identification

#### 3.1.1. Material of the Plant

*R. mucronata* was collected from the naturally growing stands of mangrove vegetation in the southern extension of the Red Sea, about 60 km south of Shalatin city along the coast (23°05′21.1″ N 35°33′08.0″ E), in July 2021. The leaves and stems were separated and cleaned with tap water and were allowed to dry for ten days in a shaded, ventilated location at a temperature of 25 °C. Every 24 h, any reduction in the weight of the leaves and stems was recorded until a fixed weight of 360 g was reached for the dry leaves and a fixed weight of 440 g was reached for the dry stems. Then, they were crushed into a fine powder [41].

#### 3.1.2. Preparation of Plant Extract

A total of 200 g of *R. mucronata* leaves and stems that had been air-dried were extracted separately using the cold percolation technique three times in 500 mL of 70% methanol for 3 days [41]. A Buchner funnel was used to filter the two methanolic extracts [41]. The filtrates were then dried in a dissector after being evaporated in a rotary evaporator at a temperature lower than 40 °C. The residues were 25 g for the leaves and 18 g for the stems. The crude methanol extracts were subjected to GC/MS analysis in order to determine their bioactive components [41].

#### 3.1.3. Phytochemical Screening

Different plant extract samples were analyzed using a gas chromatography/mass spectrometry (Thermo Scientific TRACE 1310) device (J & W Scientific). A continuous flow of 1 mL/min of helium was used as a carrier gas for the sample analysis. The oven’s temperature was ramped up from 40 °C to 280 °C at a rate of 5 °C per minute. The injection quantities were 1 µL, and the withholding period was 7.5 min. The ion source was adjusted to a temperature of 280 °C. The sample was ionized in the electron impact mode using a mass range of *m/z* 50–650 and an ionization voltage of 70 eV. The data interpretation was conducted utilizing databases from the Wiley and Nist libraries.

### 3.2. Cytotoxic Evaluation

The tissue culture laboratory at Vacsera, Dokkey, Giza, Egypt provided prostate cancer cells (PC-3), colon cancer cells (CaCo-2), breast cancer cells (MCF-7), lung cancer cells (A549), and normal human fetal lung fibroblasts (WI-38).

#### 3.2.1. Culturing

The sterility of the process was maintained using a laminar airflow cabinet. The Roswell Park Memorial Institute medium (RPMI 1640) was used to sustain the cell culture. A mixture of 1% antibiotic and antimycotic (10,000 μg/mL streptomycin sulphate, 25 μg/mL amphotericin B, and 10,000 U/mL potassium penicillin) and 1% L-glutamine were added to the medium, and 10% heat-inactivated fetal bovine serum was used as a supplement [41].

#### 3.2.2. MTT Assay

The MTT assay was employed in order to measure cytotoxicity. A purple formazan is created from the yellow MTT via mitochondrial reduction [41]. For inoculation, a 96-well microplate was filled with 1 × 10^5^ cells per well in 100 µL of Roswell Park Memorial Institute medium (RPMI 1640). The microplates were incubated at 5% CO_2_ and 37 °C for 24 h in order to develop a completely formed monolayer sheet. After the cells formed a confluent layer, the growth medium was decanted from the 96-well microplates. Dimethyl sulfoxide (0.1%) was used to dissolve the methanol extracts from the leaves and stems. The dissolved extract was serially diluted using growth medium in order to achieve the final concentrations of 156.25, 312.5, 625, 1250, 5000, and 10,000 µg/mL. The confluent cell monolayers were injected with 0.1 mL of the extract at each concentration using a multichannel pipette, and then they were dispersed throughout the 96 wells. The cells that were treated with the extracts were incubated at 37 °C and 5% CO_2_ for 24 h. Three wells were used for each extract concentration. The control cells were incubated without leaf or stem extracts. Phosphate-buffered saline (Bio Basic Canada Inc.) was used to dissolve the MTT powder in order to provide a solution with a 5 mg/mL concentration. Each well received 20 µL of the MTT solution following completion of the incubation period. A shaker (MPS-1, Biosan, London, UK) was used for mixing, which was set to 150 rpm for 5 min. The 96-well microplates were then kept for 4 h at 37 °C and 5% CO_2_. A metabolic byproduct of the MTT, called formazan, was resuspended in 200 µL of DMSO and was aggressively shaken for five minutes at 150 rpm. The optical density at 560 nm was determined using a microplate reader. A background reference wavelength of 620 nm was used to adjust the results [41]. All experiments were performed in triplicate.

#### 3.2.3. Determination of IC_50_ Values

Using GraphPad Prism version 7 software (San Diego, CA, USA), the IC_50_ values of the various concentrations of the methanol extracts of *R. mucronata* and doxorubicin (as a positive control) against CaCo-2, PC-3, MCF-7, A549, and WI-38 cell lines were computed. Equation (1) was used to determine the percentage of growth inhibition [41]:

Growth Inhibition (%) = 100 − (mean OD of individual test group/mean OD of control group) × 100(1)

#### 3.2.4. Criteria for Anticancer Effect Levels

The level of cytotoxicity of the methanol extract of *R. mucronata* was categorized using the Geran protocol and the protocol described by the National Cancer Institute (NCI) of the United States as a highly cytotoxic substance (IC_50_ < 20 µg/mL), a moderately cytotoxic substance (IC_50_ of 21–200 µg/mL), a weakly cytotoxic substance (IC_50_ of 201–500 µg/mL), or a non-cytotoxic substance (IC_50_ > 501 µg/mL) [42,43].

#### 3.2.5. Selectivity Index

The ratio of a plant extract’s IC_50_ value in a non-cancer cell line (WI-38) to its IC_50_ value in each cancer cell line is known as the selectivity index. SI values below three indicate that the extract is not selective in relation to non-cancer cells [42,43]. The selectivity indices of the methanol extract were determined using Equation (2):

(Selectivity Index = IC_50_ of the non-cancer cell line (WI-38)**/**(IC_50_ of the cancer cell line) (2)

#### 3.2.6. Microscope

The morphological structures of the cell lines were examined using a Nikon 11,881 inverted microscope at 8× at various methanol *Rhizophora mucronata* extract concentrations.

## 4. Discussion

In this study, alkaloid derivatives, such as benzo[H]quinoline-4-carboxylic acid and thieno[3,4-c]pyridine, 1,3,4,7-tetraphenyl, were identified via GC/MS for the first time in *Rhizophora mucronata*. The anticancer effect of the stems of *R. mucronata* was evaluated for the first time against lung cancer cell lines and was compared to that of the plant’s leaves. It was found that the anticancer effect of the methanol extract from the stems of *R. mucronata* against lung cancer was greater than that of the leaf extract. Additionally, the stems did not show any cytotoxicity to the normal cell lines; therefore, the stem of *R. mucronata* may have potential selectivity for lung cancer without adversely affecting normal cells. The anticancer effects of the leaf and stem extracts of *R. mucronata* were similar against breast and colon cancer cell lines.

The GC/MS data for the leaf and stem extracts of *Rhizophora mucronata* revealed different chemical compounds, including hydrocarbons, fatty acids, alkaloids, carbohydrates, and sterols. The major component of the methanol extracts was inositol, which has a similar structure to glucose. The plant increases its osmotic pressure in the cell sap by increasing the concentration of the inositol compound to 33% in the leaves and 27% in the stems. This is an internal characteristic that is considered an adaptive response to resist the high salinity tolerance of the environment [44]. On the other hand, most of the identified compounds found in the methanol extracts of the leaves and stems have been reported to have anticancer effects. For example, Benzo[H]quinoline-4-carboxylic acid is a quinoline alkaloid with a reported potential anticancer effect [26]. The anticancer and antimicrobial effects of tetradecamethyl-cycloheptasiloxane have been reported [27,28]. A fatty acid, 9,12-octadecadienoic acid, has been previously reported to have anticancer and antimicrobial effects [36,37]. Although the anticancer effects of the other two identified fatty acids, cyclopropanetetradecanoic acid, 2-octyl-, methyl ester and methyl 14 methylpentadecanoate, have not been evaluated, their antimicrobial effects have been previously reported [29,30]. Desulphosinigrin is a glucosinolate that has also been reported to have anticancer and antimicrobial effects [32,33,34]. The major identified compound in the leaf and stem extracts was inositol, which has been reported to have anticancer effects [38]. Thieno[3,4-c]pyridine, 1,3,4,7-tetraphenyl, a pyridine alkaloid, has been reported to have anticancer effects [40]. Another identified compound, 1H-purin-6-amine, N- [(3-fluorophenyl)methyl], a heterocyclic aromatic, has not been evaluated for its anticancer effects, but it has been reported to have antioxidant activity [31]. Hexasiloxane, tetradecamethyl has been reported to have antimicrobial effects [39]. The carbohydrate 3-O-Methyl-d-glucose has been reported to possess anti-inflammatory and antioxidant effects [35]. The biological activities of the alkaloid, 4-Chloro-3-methoxy-2-methylpyridine, the sterol, (5α)-cholest-1-en-19-ol, and the aromatic organic compound, alpha-tetraloxime, 5,6-dimethoxy, have not been evaluated until now (Table 1).

This study revealed that the greatest anticancer effects of both of the parts of *R. mucronata* was on the colon (CaCo-2) and breast (MCF-7) cancer cell lines. This observation agrees with the results reported by Yunos et al. [45], who found that Epi-catechin, 4-O-caffeoyl quinic acid, 5-Ocaffeoyl quinic acid, and procyanidin B2 isolated from *R. mucronata* showed strong to moderate anticancer effects on colorectal (HT29) and breast (T47D) cancer cell lines. These results also agree with existing studies on the polyisoprenoids from *R. mucronata* leaves that induce apoptosis in WiDr colon cancer cells by reducing the expression of Bcl-2 and cyclin D1, consequently causing cell cycle arrest [25]. Furthermore, our results show that the leaves possess anticancer effects on breast (MCF-7) cancer cell lines, with an IC_50_ value of 158 ± 10 μg/mL. These results are consistent with those in the literature, where quinizarin isolated from the methanol extract of *R. mucronata* leaves demonstrated anticancer effects against breast (MDA-MB231) cancer cell lines [24]. Other anticancer effects of *R. mucronata* leaves have been reported in the literature [23,46] on cervical (Hela) and myeloid leukaemia (HL-60) cancer cell lines. Interestingly, the anticancer effects of *R. mucronata* was different between the leaf and stem extracts on lung (A549) cancer cell lines, which showed IC_50_ values of 376 ± 9 μg/mL and 155 ± 10 μg/mL, respectively. However, there was no difference between the anticancer effects of the leaves and stems of *R. mucronata* on prostate (PC-3) cancer cell lines, which were both ineffective with IC_50_ values of 480 ± 14 μg/mL and 294 ± 3 μg/mL, respectively. Our results also show that neither the leaves nor the stems of *R. mucronata* exhibited cytotoxicity against normal human fetal lung fibroblasts (WI-38), which showed IC_50_ values of 932 ± 30 μg/mL and 629 ± 3 μg/mL, respectively (Table 2). Moreover, the leaf and stem extracts had selective cytotoxicity towards the studied cancer cells, with the exception of prostate cancer cells (PC-3), and the stem extract’s selectivity for lung cancer (A549) was greater than that of the leaf extract when compared to normal cells (Table 3). Further investigations are necessary in order to explore the phenolic and flavonoid compounds present in *R. mucronata* and to estimate their anticancer effects on several cancer cell lines.

## 5. Conclusions

The leaves and stems of *R. mucronata* were analyzed via a phytochemical analysis, and both contained similar constituents, including benzo[H]quinoline-4-carboxylic acid, 1H-purin-6-amine, n- [(3-fluorophenyl)methyl], inositol, 3-O-methyl –d-glucose, and desulphosinigrin. However, the leaves contained active compounds, including 6-chloro-3-methoxy-2-phenylindole, cycloheptasiloxane, tetradecamethyl, cyclopropanetetradecanoic acid, 2-octyl-, methyl Ester, methyl 14 methylpentadecanoate, 9,12-octadecadienoic acid, and (5α)-cholest-1-en-19-ol. Furthermore, the stems contained active compounds, including alpha-Tetraloxime, 5,6-dimethoxy, hexasiloxane, tetradecamethyl, and thieno[3,4-c]pyridine, 1,3,4,7-tetraphenyl. In this study, the anticancer effects of both the leaves and stems of *R. mucronata* plants were evaluated against different cancer cell lines. Our results show that the leaf and stem extracts possess greater anticancer effects against colon and breast cancer compared to prostate cancer, and that the stems of *R. mucronata* have a greater anticancer effect against lung cancer than the leaves. The presence of the diverse variety of constituents identified via the phytochemical analysis may explain the anticancer effects of the leaf and stem extracts against different cancer cell lines. In the future, these constituents may be employed as monotherapy or in conjunction with other agents as treatments for a variety of medical conditions, particularly since normal cells are not adversely affected by the cytotoxic activities of either parts of the *R. mucronata* plant.

## Figures and Tables

**Table 2:** Cytotoxic effects of methanol extract from *R. mucronata* against cancer cell lines.

		^a^ IC_50_ (µg/mL)	
Cell Lines	*R. mucronata* Leaves	*R. mucronata* Stems	Doxorubicin (Positive Control)
^b^ CaCo-2	127 ± 4 ***	107 ± 6 **	83 ± 1
^c^ PC-3	480 ± 14 ***	294 ± 3 ***	79 ± 4
^d^ MCF-7	158 ± 10 ***	138 ± 3 ***	80 ± 3
^e^ A549	376 ± 9 ***	155 ± 10 ***	90 ± 5
^f^ WI-38	932 ± 30 ***	629 ± 3 ***	50 ± 5

^a^ IC_50_: the half-maximal inhibitory concentration; ^b^ human colon cancer (CaCo-2); ^c^ prostate cancer (PC-3); ^d^ human breast cancer (MCF-7); ^e^ lung cancer (A549); ^f^ normal human fetal lung fibroblast (WI-38). The findings are shown as means ± standard deviations. ** *p* = 0.001, and *** *p* = 0.0001 in comparison to doxorubicin. The *R. mucronata* stem and leaf extracts and doxorubicin were compared using one way analysis of variance (ANOVA) and then Dunnett’s multiple comparisons test.

**Table 3:** Selectivity index values of *R. mucronata* methanol extracts from leaves and stems for CaCo-2, PC-3, MCF-7, and A549 cancer cells.

	^a^ SI			
Extract	^b^ CaCo-2	^c^ PC-3	^d^ MCF-7	^e^ A549
Leaves	7.3	2	5.8	2.4
Stems	5.8	2.1	4.5	4

^a^ SI: selectivity index; ^b^ human colon cancer (CaCo-2); ^c^ prostate cancer (PC-3); ^d^ human breast cancer (MCF-7); ^e^ lung cancer (A549). The selectivity index (SI) is defined as the ratio of IC_50_ values for normal human fetal lung fibroblast (WI-38) divided by the IC_50_ values for each cancer cell line. Values greater than 3 represent compounds that are preferentially active against cancer cells compared to non-cancer WI-38 cells.

## Data Availability

Not applicable.
